# Multiple myeloma with intracranial and spinal intradural metastasis: A case report

**DOI:** 10.37796/2211-8039.1079

**Published:** 2020-09-01

**Authors:** Wei-Lin Hsu, Jeng-Hung Guo, Ying-Ying Hung, Der-Yang Cho, Der-Cherng Chen

**Affiliations:** a Department of Neurosurgery, China Medical University Hospital, Taichung City, Taiwan; b Executive Health Management Center, Cheng Ching Hospital, Taichung City, Taiwan; c Graduate Institute of Immunology, China Medical University, Taichung City, Taiwan

**Keywords:** hydrocephalus, intracranial, intradural, multiple myeloma, spine

## Abstract

Multiple myeloma is a hematopoietic cancer that is multicentric and most commonly involves the spine. Multiple myeloma with extraosseous and intradural involvement is an extremely rare condition. Here we present a rare case of spinal multiple myeloma with intracranial and spinal intradural metastasis causing lumbar spinal nerve compression. We present a 60-year-old woman with progressive weakness of the lower limbs for several weeks. Spinal magnetic resonance imaging (MRI) showed a leptomeningeal tumor with nodularity spreading within the cauda equina. Examination of the brain using MRI showed a lytic skull bone lesion and leptomeningeal enhancement. The patient underwent L3-5 laminectomy. Immunohistological staining confirmed a diagnosis of multiple myeloma of the IgA kappa subtype. After surgery, the patient underwent chemotherapy and rehabilitation exercises. Multiple myeloma has a median survival of 2.5 years, while 75% of patients with spinal involvement die within 1 year of diagnosis. Unfortunately, our patient died 3 months after the diagnosis of multiple myeloma with spinal and intracranial involvement. Intracranial and spinal intradural multiple myeloma invasions are quitely rare. Spine biopsies and cerebrospinal fluid cytology can aid in the diagnosis. Early surgical decompression is necessary, especially when neurological deficits occur.

## 1. Introduction

Multiple myeloma is one of the most common hematological malignancies involving the spine. The disease is a clonal B-cell malignancy characterized by proliferation and accumulation of B-lymphocytes and plasma cells in the bone marrow. It subsequently breaks through cortical bone to invade the surrounding tissues [1]. Neurological compression occurs in approximately 5% of patients with multiple myeloma. Spinal cord compression is mostly caused by vertebral body collapse or extradural extension of tumor tissue from the adjacent vertebra [2,3]. Here we present a rare case of spinal multiple myeloma with intracranial and spinal intradural metastasis causing lumbar spinal compression.

## 2. Case report

**Fig. 1:**
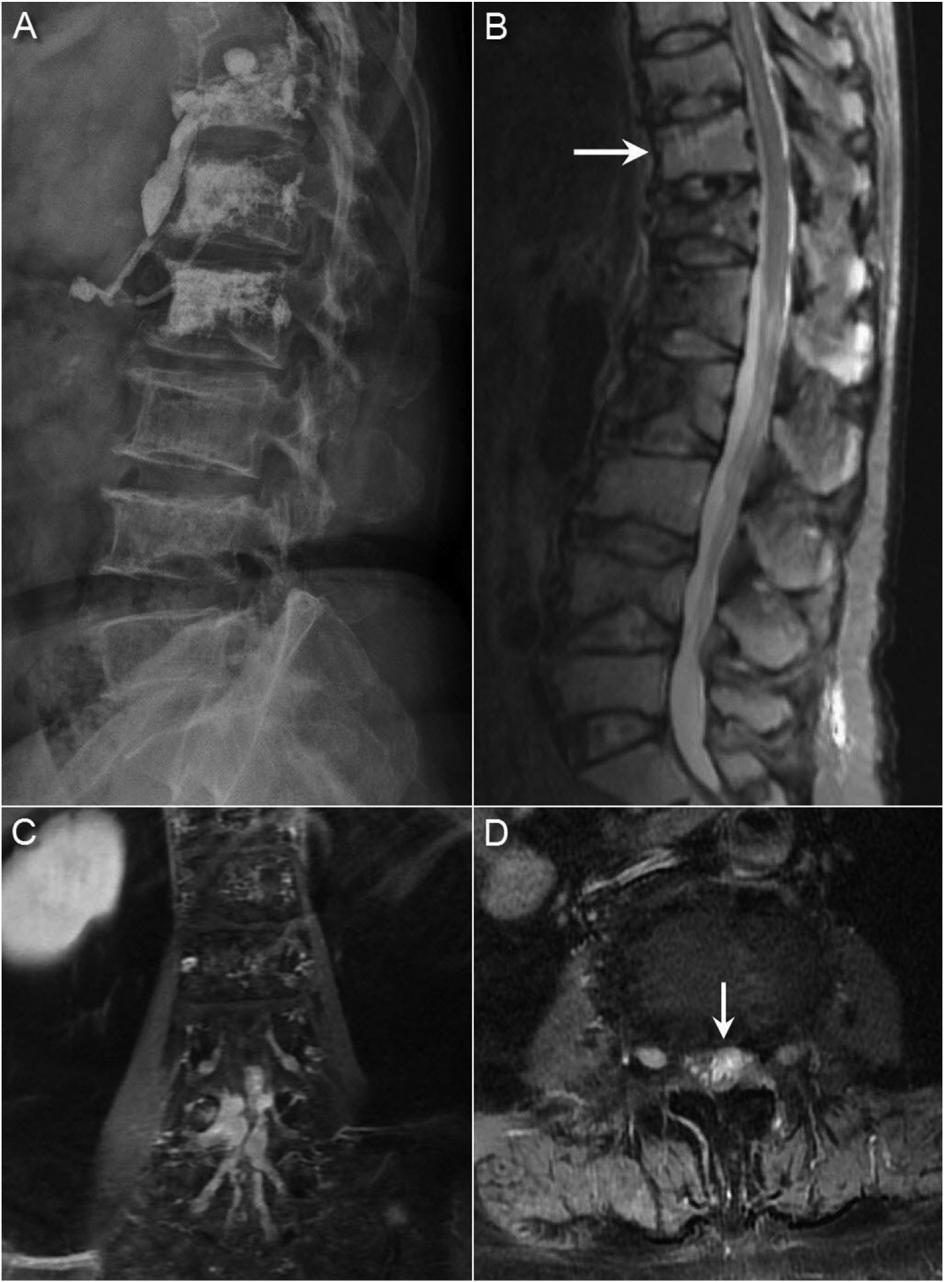
(A) Radiographs showing multiple compression fractures and a moth-eaten appearance of multiple vertebral bodies (B) Sagittal short T1 inversion recovery (STIR) sequence in the spinal MRI showing a new T11 compression fracture and multiple prior compression fractures. Coronal (C) and axial (D) T1-weighted MRI with gadolinium enhancement showing a diffuse leptomeningeal tumor spreading with extensions into the bilateral L4 and L5 neural foramen and nodularity of the cauda equina.

A 60-year-old woman, with a history of hypertension, presented with a thoracolumbar spinal compression fracture suffering from progressive back pain with numbness below the T10 sensory dermatome for days. Neurological examination found a normal bilateral plantar response. Spinal magnetic resonance imaging (MRI) revealed a new T11 compression fracture (Fig. 1A and B). The patient was scheduled for vertebroplasty to treat the new osteoporotic compression fracture, and reduced the patient's back pain significantly.

Three months later, the patient's back pain worsened and became refractory to medication. Laboratory studies revealed a hemoglobin count of 6.9 g/dL, a white blood cell count of 6430/mm^3^, and a platelet count of 92000/mm^3^. Serum liver and renal function values were within normal limits. The patient was referred to the hematological outpatient department where a bone marrow biopsy confirmed the diagnosis of multiple myeloma. Upon microscopy analysis, the marrow exhibited marked hypercellularity (90–95%) containing a diffuse infiltration of atypical plasma cells and plasmablasts with marked cellular pleomorphism and prominent nucleoli. The normal hematopoietic cells were markedly depleted.

Immunohistochemical analysis showed that the plasma cells were positive for kappa light chain and CD138, but negative for lambda light chain. Multiple myeloma of the plasmablastic type was considered. The serum IgG level was 317 mg/dL (range 751–1560 mg/dL), IgA 7710 mg/dL (range 82–453 mg/dL), IgM 6.02 mg/dL (range 46–304 mg/dL), kappa light chain 5000 mg/dL (range 629–1350 mg/dL), lambda light chain 122 mg/dL (range 313–723 mg/dL), lactate dehydrogenase (LDH) 238 IU/L (range 98–192 IU/L), and β-2 microglobulin 5156 ng/mL (range 1010–1730 ng/mL).

The patient was diagnosed with multiple myeloma of the IgA kappa subtype, and a chemotherapy regimen comprising zoledronic acid and bortezomib was administered.

**Fig. 2:**
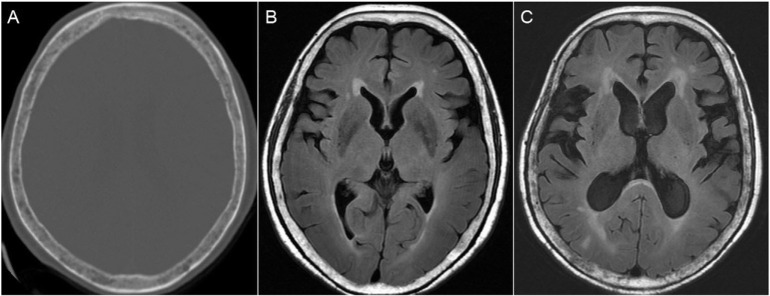
(A) Axial brain CT images showing osteolytic lesions in the skull. Brain MRI of the patient at presentation (B) and four months later (C) showing progressive hydrocephalus and prominent skull bone lytic lesions.

Initially, limb strength was normal, but progressive weakness in the lower limbs was observed with grade IV strength after 2 years. The patient was unable to walk independently. Repeat spinal MRI showed a diffuse leptomeningeal tumor spreading with extensions into the bilateral L4–L5 neural foramen and the L4 body (Fig. 1C and D). Because of leptomeningeal spreading was identified, whole neuroaxis surveillance was indicated. Head computed tomography (CT) examination of the brain showed multifocal osteolytic lesions of the skull, which was compatible with the prior diagnosis of multiple myeloma (Fig. 2A). The patient was admitted under the impression of spinal multiple myeloma with intracranial and intradural metastasis.

During L3-5 laminectomy, a soft, grayish-white tumor was found adherent to the dura. Additional soft and white-colored tumors were found posterolaterally and extending along the L4 nerve roots into the neural foramen. The tumor was partially removed with microscopic assistance.

**Fig. 3:**
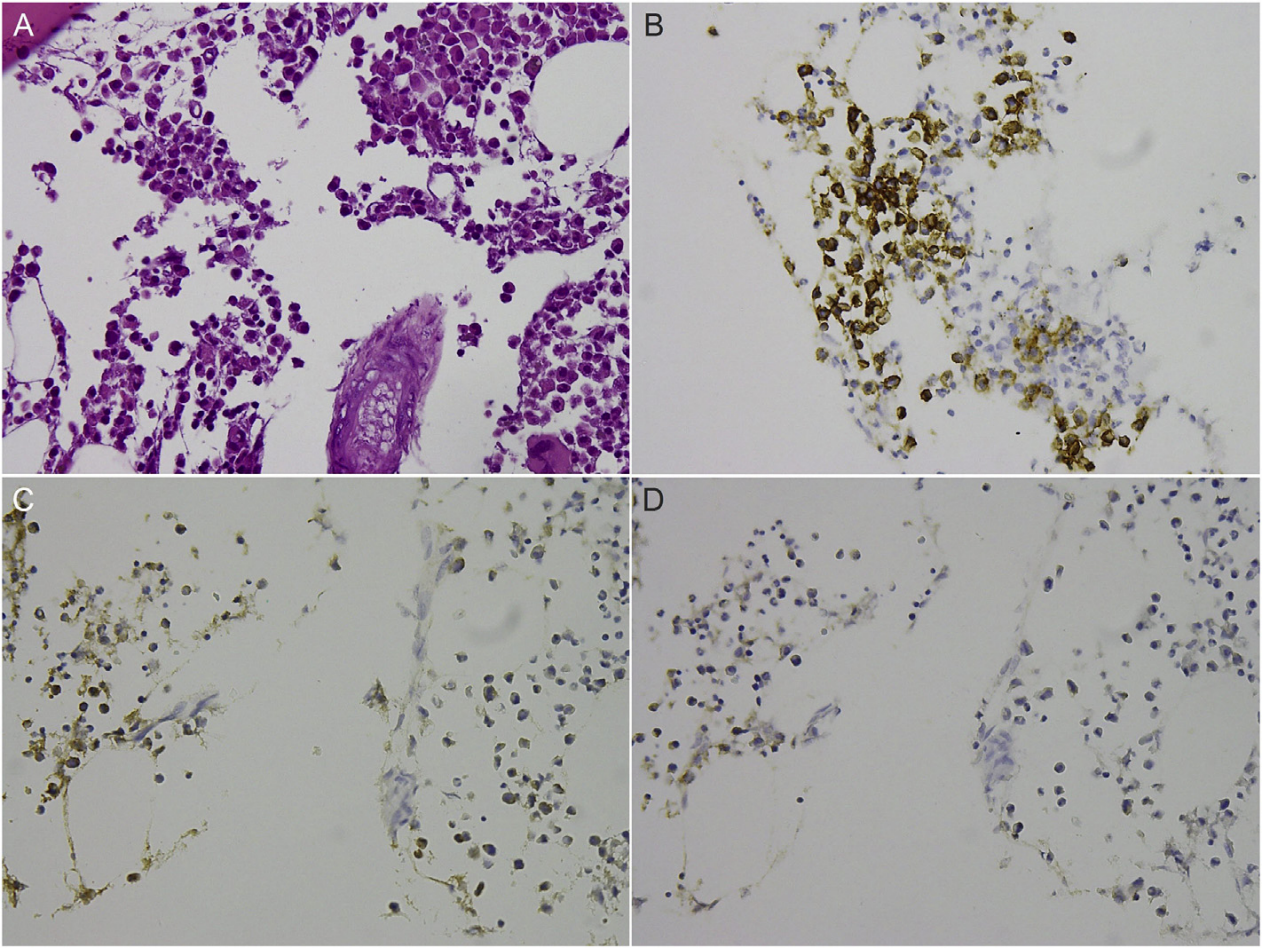
(A) Histological examination of the myeloma showing aggregation of atypical plasma cells with mild cellular pleomorphism, abundant cytoplasm, and binucleation in some of the cells (Hematoxylin and eosin, 400X). Immunohistochemical analysis results of the plasma cells showing positive staining for CD138 (B) and kappa light chain (C), but negative staining for lambda light chain (D).

The histopathological examination of the tumor revealed aggregations of atypical plasma cells with mild cellular pleomorphism. Some cells were binuclear with abundant cytoplasm, and a few cells were observed to be undergoing mitosis. Immunohistochemical staining showed the plasma cells to be positive for CD138 and kappa light chain, but negative for lambda light chain (Fig. 3). This was consistent with the prior diagnosis of multiple myeloma of the kappa subtype. The patient was subsequently treated with a respiratory training program and rehabilitation exercises. Unfortunately, she died of pneumonia with multiple organ failure two months after the surgery.

## 3. Disscussion

Osteoporotic and pathological vertebral compression fractures are a major cause of morbidity and inferior quality of life among the elderly. Percutaneous vertebroplasty is an effective procedure to treat pain and reduce the risk of developing complications due to long-term immobilization. However, the incidence of new compression fractures after the initial percutaneous vertebroplasty is 11–52% [4]. Further, 67% of new compression fractures are adjacent to the previously treated vertebral level [5]. The risk factors affecting subsequent fractures after vertebroplasty include old age, osteoporosis, long-term steroid use, the augmented stiffness of the treated vertebra, cement leakage into the intervertebral disc space, and other poorly defined underlying diseases [4,5]. Some metastatic tumors, such as lymphoma, Ewing's sarcoma, and multiple myelomas may cause vertebral body collapse resembling osteoporotic compression fractures. Due to this similarity, it can be difficult to identify osteolytic tumors in osteoporotic patients. The radiography images of our patient showed a moth-eaten appearance in multiple vertebral bodies (Fig. 1A). We propose that spine biopsies should be performed earlier when a bone lesion is suspected [6].

Multiple myeloma, also known as plasma cell myeloma, is a cancer of plasma cells, usually accumulating in the bone marrow. It accounts for 1% of malignant tumors and 10–15% of hematopoietic neoplasms. Complications include loss of bone with cortical bone lysis, with the spine being one of the most common sites of pathological fracture [1,3]. The median age at diagnosis is 63 years, with only 12% of cases reported in patients younger than 50 years and 2% of cases in those younger than 40 years. Furthermore, the incidence is extremely rare in younger populations where 0.18% to 0.3% of cases were observed in patients younger than 30 years old [7,8].

Extraosseous myeloma is an uncommon and aggressive form of the disease, accounting for a cumulative incidence of 4.6% of patients with multiple myeloma [9]. The common locations of extra-osseous myeloma are the mucosa of the upper airway, including the oropharynx, nasopharynx, nasal cavity, paranasal sinuses, and the gastrointestinal tract [9,10]. The central nervous system may be involved through adjacent bone lesions or by spreading through the blood with parenchymal infiltration [3,11]. The median interval from diagnosis of multiple myeloma to diagnosis of central nervous system (CNS) myeloma is around 6–18 months [11].

Extraosseous epidural myeloma without local vertebral body involvement is very rare. Only 7 cases of isolated extraosseous epidural myeloma are reported in the literature [3,10,12]. The differential diagnoses include neoplasms or inflammatory conditions, such as meningioma, neurinoma, lymphoma, solitary amyloidoma, hemorrhagic vascular lesions, and epidural abscess [3,10,12]. However, it is quite difficult to distinguish these conditions on the basis of imaging signal intensity and morphology prior to surgical diagnosis.

Intradural myeloma is extremely rare. The standard diagnosis of leptomeningeal carcinomatosis (LC) is the identification of tumor cells in the cerebrospinal fluid (CSF). However, CSF cytology has a false negative rate of 10–15% [13]. Hayakawa et al. [14] reported a case of multiple myeloma in a 55-year-old man with progressive left-sided pain in the back and arm. He had achieved hematological complete remission after chemotherapy and radiotherapy, but 21 months later, he developed an intradural recurrence of multiple myeloma in his cauda equina and cerebrum. There were plasma cells detected in the CSF as well as IgG-lambda type M-protein. Unfortunately, in our case there was no associated CSF evidence.

LC is a devastating condition and it occurs in approximately 1–5% of all patients with cancers [13,15]. Hydrocephalus may result as a complication of LC due to the obstruction of CSF by a tumor mass or from the dissemination of metastatic cells in the subarachnoid space inducing CSF malabsorption [16]. Further, brain MRI in our case showed lytic skull bone lesions and leptomeningeal enhancement of the cerebral cortex. The hydrocephalus also progressed in comparison with the previous brain MRI performed 4 months ago (Fig. 2B and C). We propose that multiple myeloma with CNS myelomatosis or dural-based lesions may spread down-ward into the spinal cord through CSF circulation and cause obstructive hydrocephalus.

Treatment of extraosseous multiple myeloma includes surgical resection and radiotherapy of 4000 cGy over 4 weeks. Cranial and spinal radiation may improve survival from 1 month to 5 months for those who have an initial response to therapy. The response of chemotherapy to extraosseous myeloma is very poor [3,10,11].

Multiple myeloma has a median survival of 2.5 years, while it has been reported that 75% of patients with spinal involvement die within 1 year of diagnosis [1,7,8]. The most significant predictive factor for survival among all clinical parameters is β-2 microglobulin level. The increased serum β-2 microglobulin level of 5156 ng/mL and serum albumin of 3.0 g/dL in this case corresponds to a classification of stage II according to the international staging system (ISS) for multiple myeloma, with a median survival of 44 months [17]. Furthermore, the elevated serum LDH level implies an increased aggressiveness of the disease and suggests a high proliferation rate and/or the presence of tumor mass, in particular extramedullary and extraosseous disease. The elevated serum LDH level of 238 IU/L in our case was associated with shorter overall survival [18]. Unfortunately, our patient died 3 months after the diagnosis of multiple myeloma with spinal involvement.

## 4. Conclusion

Although multiple myeloma with extraosseous and intradural involvement is an extremely rare condition, earlier detection and the ability to distinguish it from diseases with similar presentation is required, especially when neurologic deficits occur. Spine biopsies and CSF cytology can be helpful in the diagnosis. Early decompression of the tumor may prevent neurological deterioration and preserve better quality of life.

## Abbreviations

CNS: central nervous system
CSF: cerebrospinal fluid
CT: Computed tomography
LC: leptomeningeal carcinomatosis
LDH: lactate dehydrogenase
MRI: magnetic resonance imaging
STIR: Sagittal short T1 inversion recovery
